# Exploring barriers, facilitators, and preferences for the uptake of contraceptive methods among adolescents aged 15–19 years in Neno district, Malawi: a qualitative study

**DOI:** 10.1080/26410397.2026.2702243

**Published:** 2026-07-13

**Authors:** Abraham Ngwira, Faith C. Adigwe, Maxwell Mhlanga, Matamando Mwendera, Moses Banda Aron, Fabien Munyaneza

**Affiliations:** a Alumnus, Centre for Gender Equity, University of Global Health Equity, Kigali, Rwanda. *Correspondence:* abrahamngwira@gmail.com, abrahamngwira733@gmail.com; b Alumnus, Centre for Gender Equity, University of Global Health Equity, Kigali, Rwanda; c Associate Professor, Centre for Gender Equity, University of Global Health Equity, Kigali, Rwanda; d Research Officer, Partners in Health Malawi, Neno, Malawi; e Research and Impact Manager, Partners in Health Malawi, Neno, Malawi; f Monitoring Evaluation Informatics & Research Director, Partners in Health Malawi, Neno, Malawi

**Keywords:** intersectionality, culture, religion, protection, consequences, family, planning, adolescents

## Abstract

Adolescent contraception is important in reducing sexually transmitted infections and teenage pregnancies. However, its uptake remains low at 10% in Neno district, despite various interventions promoting free access. A phenomenological qualitative study was carried out in the district to explore the barriers, facilitators and preferences for the uptake of contraceptive methods among adolescents aged 15–19. Five focus group discussions and 18 in-depth interviews were carried out with adolescents, parents, healthcare workers, community leaders and youth volunteers. Data was collected in Chichewa, translated into English, and analysed using thematic analysis. Three key themes appeared: (1) a climate of restriction: stigma, misinformation, and systemic barriers; (2) agency and support: navigating pathways to contraceptive uptake; and (3) the paradox of acknowledged risk and sustained resistance. Adolescents in Neno faced socio- cultural barriers, health facility limitations and informational gaps, alongside individual and external facilitators like education and peer influence. Adolescent choices were shaped by ease of use, sexual desires and economic hardships. Therefore, an integrated approach involving healthcare workers, policymakers, and non-governmental organisations (NGOs) is crucial to improving contraceptive uptake in Neno.

## Plain language summary

Adolescent contraception is crucial. It helps prevent sexually transmitted infections and teen pregnancies. Partners in Health implemented a 5-year project in Neno, Malawi. The goal was to improve access to free contraception for young people. Still, only 10% of adolescents in the district are using these services. This study looked at what contraceptive methods young people aged 15–19 prefer. It also looked into what helps or hinders them to obtain these products. We carried out 5 focus group discussions and 18 in-depth interviews. Young people, parents/guardians, healthcare workers, youth workers and community leaders took part. We used thematic analysis to analyse the data from the discussions and interviews. The study showed an interaction of barriers and facilitators. These were at the individual, interpersonal, community, and health facility levels. Socio-cultural factors, information gaps, and health facility issues reduced uptake. Young people's intention to practise safe sex, and support from their peers, improve uptake. Youth-friendly sexual and reproductive health services support young people's contraceptive choices. Three key themes appeared: (1) Stigma, misinformation and service barriers are widespread. (2) Finding ways to improve contraceptive uptake is needed. (3) The community understands the risks of unsafe sex but resists contraceptive protection. Collaborative teamwork is crucial. Healthcare workers, policymakers, and NGOs should work together to improve young people's use of contraception.

## Résumé

La planification familiale est importante chez les adolescents pour réduire les infections sexuellement transmissibles et les grossesses précoces. Néanmoins, son utilisation reste faible, à 10%, dans le district de Nemo, en dépit de plusieurs interventions favorisant un accès gratuit. Une étude qualitative phénoménologique a été réalisée dans ce district afin d’explorer les obstacles, les facteurs facilitant l’accès et les préférences des adolescents âgés de 15 à 19 ans en matière d’utilisation des méthodes de planification familiale. Cinq groupes de discussion et 18 entretiens approfondis ont été menés auprès d’adolescents, de parents, d’agents de santé, de dirigeants communautaires et de jeunes bénévoles. Les données recueillies en chichewa, ont été traduites en anglais et analysées thématiquement. Trois thèmes principaux sont apparus: (1) un climat de restriction: stigmatisation, désinformation et obstacles systémiques; (2) l’autonomie et le soutien: les démarches pour accéder à la planification familiale; et (3) le paradoxe du risque reconnu et de la résistance persistante. À Neno, les adolescents sont confrontés à des obstacles socioculturels, aux limitations des centres de santé et à un manque d’information, ainsi qu’à des facteurs individuels et externes facilitant l’accès à la planification familiale, comme l’éducation et l’influence des pairs. Leurs choix sont déterminés par la facilité d’utilisation, leurs désirs sexuels et les difficultés économiques. Par conséquent, une approche intégrée réunissant les agents de santé, les décideurs et les organisations non gouvernementales (ONG) est essentielle pour améliorer le recours à la planification familiale à Neno.

## Resumen

La planificación familiar en la adolescencia es importante para reducir las infecciones de transmisión sexual y los embarazos adolescentes. Sin embargo, a pesar de diversas intervenciones para promover el acceso gratuito a la planificación familiar, su adopción en el distrito Neno continúa siendo baja, de 10%. Se realizó un estudio cualitativo fenomenológico en el distrito para explorar las barreras, los facilitadores y las preferencias para la adopción de métodos de planificación familiar entre adolescentes de 15 a 19 años. Se realizaron 5 grupos focales y 18 entrevistas a profundidad con adolescentes, padres, trabajadores de salud, líderes comunitarios y jóvenes voluntarios. En Chichewa, se recolectaron datos, que se tradujeron al inglés y se analizaron utilizando análisis temático. Surgieron tres temas clave: (1) un clima de restricción: estigma, información errónea y barreras sistémicas; (2) agencia y apoyo: navegando las vías a la adopción de PF; y (3) la paradoja de riesgo reconocido y resistencia continua. Las adolescentes en Neno enfrentaban barreras socioculturales, limitaciones de los centros de salud y brechas informativas, así como facilitadores individuales y externos como la educación y la influencia de pares. La toma de decisiones de las adolescentes era guiada por la facilidad de uso, deseos sexuales y dificultades económicas. Por ello, para mejorar la adopción de la planificación familiar en Neno es fundamental aplicar un enfoque integrado que incluya a trabajadores de salud, formuladores de políticas y organizaciones no gubernamentales (ONG).

## Introduction

Globally, adolescents aged 15–19 recorded an adolescent birth rate of 41.3 births per 1000 women in 2023.^1^ This high fertility rate underscores a critical gap in realising sexual and reproductive health rights (SRHR), which include the right to access information and services to make autonomous, informed decisions about one’s body and fertility.^2,3^ The inability of adolescents to exercise these rights has profound implications, driving cycles of poor health, gender inequality, and socioeconomic marginalisation.^4^ Only half of adolescents aged 15–19 are estimated to have their demand for modern contraception satisfied, leaving millions with an unmet need that constitutes a direct breach of their reproductive rights.^1,5^ The consequences are severe: in low- and middle-income countries (LMICs), pregnancy and childbirth complications are a leading cause of death for adolescent girls, and their infants face significantly higher mortality risks.^6,7^ These outcomes highlight the systemic failure to uphold adolescents’ rights to life, health, and bodily autonomy.

Sub-Saharan Africa (SSA) bears the highest adolescent fertility rate globally.^1^ While the global adolescent birth rate declined between 1990 and 2015, progress in SSA has been slow and uneven.^1^ This regional burden is a manifestation of intersecting rights denials, including lack of access to youth-friendly, confidential services, pervasive gender norms that limit girls’ agency, and stigma surrounding adolescent sexuality.^8,9^ Research consistently identifies unmet need for contraception, driven by structural and socio-cultural barriers, as the primary driver of adolescent pregnancy in the region.^10,11^ A robust body of literature, framed through both public health and rights-based lenses, has elucidated these barriers across socio-ecological levels. At the individual and interpersonal level, key obstacles include lack of comprehensive knowledge about sexual and reproductive health, fear of side effects, low self-efficacy, and opposition from male partners or parents.^12,13^ Community and societal barriers encompass stigmatising attitudes, restrictive gender norms, and religious or cultural disapproval of adolescent contraceptive use.^9,14^ Finally, health system barriers such as judgmental provider attitudes, lack of privacy, and stock-outs of preferred methods directly impede the right to accessible, acceptable, and quality health services.^7,14^

Malawi, a sub-Saharan African country where 51% of the population is under 18, exemplifies these challenges^15^ Approximately 32% of adolescent girls aged 15–19 have begun childbearing, a national average that masks significant subnational disparities.^16^ Neno district, a rural setting, reports a critically high unmet need for contraception at 52% among youth.^17^ Despite contraceptive services being nominally available in all health facilities, adolescent utilisation remains remarkably low at 10%, contributing to high rates of teenage pregnancy, unsafe abortion, and school dropout.^17^ This gap between service availability and utilisation points to a rights-based deficit: services are not being delivered in a manner that is adolescent-responsive, confidential, or free from discrimination, as mandated by international human rights frameworks.^3^

Existing research on adolescent contraception barriers and facilitators, while extensive, reveals contextual and methodological gaps that this study addresses. Prior work in SSA has often excluded male adolescents, whose attitudes and involvement are critical to understanding and addressing rights-related dynamics like negotiation and shared decision-making.^3,5^ Studies frequently focus on school-going or urban populations, limiting understanding of the compounded vulnerabilities faced by out-of-school adolescents in rural areas like Neno.^13,18^ Furthermore, while several studies list barriers, fewer employ a comprehensive socio-ecological or explicit rights-based framework to analyse how multi-level factors interact to restrict adolescents’ reproductive autonomy.^19,20^

This study, therefore, adopts an explicit rights-based lens to investigate the barriers, facilitators, and preferences regarding contraceptive use among adolescents aged 15–19 in Neno district, Malawi. By intentionally including both male and female adolescents and utilising a socio-ecological framework, it seeks to understand the intersecting factors that impede or enable the realisation of adolescents’ SRHR. The findings aim to inform the design of rights-affirming, adolescent-responsive contraceptive services that are not only accessible but also acceptable and equitable, thereby contributing to the fulfilment of adolescents’ fundamental rights to health, information, and self-determination.

## Methods

### Study design and setting

This phenomenological qualitative study explored the barriers, facilitators, and preferences related to contraception among adolescents aged 15–19 in Neno district, Malawi. The study design was chosen to deeply understand the lived experiences of adolescents and key stakeholders, focusing on how socio-ecological factors enable or impede the realisation of adolescents’ SRHR. Neno, a rural district in Malawi's southern region, is served by 14 health facilities offering contraceptive services through the Ministry of Health's Youth-Friendly Health Services programme. A rights-based approach was embedded in the study design and procedures, ensuring the principles of participation, non-discrimination, accountability, and transparency were upheld. This involved obtaining informed assent from adolescents and consent from parents/guardians for minors, ensuring confidentiality, and framing questions around adolescents’ entitlements to information, services, and bodily autonomy.

### Study population and sampling

The study purposively recruited male and female adolescents aged 15–19 and key stakeholders who influence adolescent contraception access. This inclusive sampling strategy, which specifically included often-overlooked male adolescents, was guided by the right to participation and the recognition that realising SRHR requires understanding the perspectives of all actors in adolescent social ecology. Adolescents were eligible if they were sexually active and had lived in Neno for ≥6 months. Stakeholders included parents/guardians, religious leaders, traditional leaders, Youth Community-Based Distribution Agents (YCBDAs), and healthcare workers. Participants with cognitive impairment were excluded. We conducted 18 in-depth interviews (IDIs) (10 adolescents, three religious leaders, one traditional leader, two YCBDAs, two healthcare workers) and 4 focus group discussions (FGDs) with parents/guardians (*n* = 38 total FGD participants). Recruitment continued until thematic saturation was reached, assessed through iterative data analysis and team debriefs where no new substantive themes emerged from subsequent interviews. Of the 10 adolescent IDI participants, 4 were male and 6 were female; 3 were aged 15–17 (minors) and 7 were 18-19; 6 were in-school and 4 were out-of-school (defined as not enrolled in formal education at the time of the study, regardless of reason).

### Conceptual and analytical framework: a rights-based socio-ecological model

**Figure 1.**
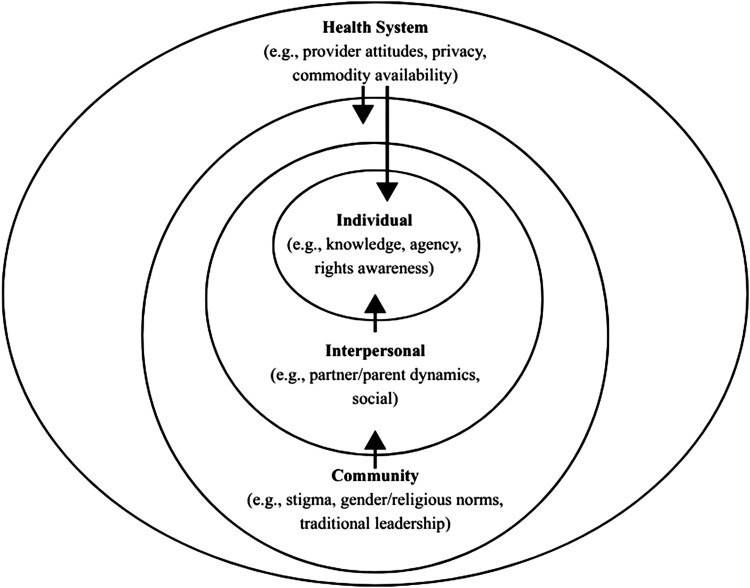
Conceptual framework of a rights-based socio-ecological model for adolescent contraceptive uptake adapted from Aventin and Gordon, 2021^6^

Data collection and analysis were guided by a rights-based adaptation of the socio-ecological model (SEM).^3,20^ The SEM was selected because it provides a structured, multi-level lens to analyse how interconnected factors at individual, interpersonal, community, and health system levels collectively shape health behaviours and outcomes: in this case, adolescent contraceptive use.^20^ Crucially, we operationalised this model through a rights-based perspective, interpreting barriers as violations or gaps in rights fulfilment and facilitators as enablers of rights realisation at each level. For instance, a community-level barrier such as stigma was analysed not just as a social norm but as a violation of the right to non-discrimination and privacy. The framework (see Figure 1) explicitly linked each level to specific SRHR principles (e.g. individual agency linked to autonomy; health system factors linked to availability, accessibility, acceptability, and quality of services).^20,21^ This adapted framework directly informed the development of our semi-structured interview guides and the thematic codebook.

Figure 1 below depicts an interactive SEM model, showing how different levels affect or complement each other in a complex way to shape the vulnerability of adolescents. Pervasive community-level stigma and religious norms directly empower interpersonal-level actors (parents, partners) to oppose adolescent contraceptive use, which in turn feeds individual-level internalised shame and fear. This creates a cascade of restriction from the broad social sphere down to the individual's decision-making. Health system-level failures such as judgmental providers and lack of privacy do not exist in a vacuum. They actively validate and reinforce community-level stigma (by making shaming public), while simultaneously eroding individual-level trust and confidence in seeking care. The system thus institutionalises social barriers.

### Data collection, tools, and reflexivity

Data were collected from May to June 2024. The semi-structured IDI and FGD guides were developed in English, translated into Chichewa, and pre-tested for cultural appropriateness and clarity. Guides included open-ended questions and probes exploring contraception experiences, perceived rights to access services, and multi-level influences. They are available as supplementary files. Data collectors used active listening and empathetic probing, and discussions were held in private, comfortable venues of the participants’ choice (homes, community centres). Interviews lasted 25–30 minutes and FGDs 40–50 minutes; this duration was adequate to establish rapport and explore topics in depth while respecting participant time and comfort, as confirmed during piloting.

Four data collectors (two male, two female all holding bachelor’s degrees with qualitative research experience) received a 5-day training. Training content included research ethics, the rights-based SEM framework, techniques for non-judgmental engagement with adolescents, and mitigation of power dynamics. The use of young, gender-matched data collectors was a deliberate strategy to reduce hierarchical barriers and encourage open discussion of sensitive topics.^22^ Researcher reflexivity was maintained through daily debriefs. The research team acknowledged their positions as health professionals and/or outsiders to the community; these potential biases were mitigated by bracketing assumptions during analysis and using a structured, framework-driven analytical process.

### Data management and analysis

All interviews were audio-recorded, transcribed verbatim in Chichewa, and translated into English by a professional translator. To ensure conceptual fidelity and adhere to principles of accountability in our rights-based approach, a subset of transcripts was back-translated and verified with participants (member checking) where feasible. Analysis was conducted in Dedoose using a hybrid deductive-inductive thematic approach.^23^ IDI and FGD transcripts were analysed together to triangulate perspectives across participant types, though we remained attentive to the unique dynamics of each method.

Guided by our framework, we first applied a deductive codebook based on the SEM levels and rights concepts. Through iterative reading, inductive codes capturing emergent themes were added. Two principal investigators coded transcripts independently; discrepancies were resolved through discussion and, if needed, consultation with a third researcher. Coded data were collated into themes, which were then interpreted through the integrated lens of the SEM and rights-based principles to move beyond description to an interpretive analysis of how multi-level factors interact to constrain or enable SRHR. This study followed the Consolidated Criteria for Reporting Qualitative Research (COREQ) checklist.^24^

### Ethical considerations

The study received ethical approval from the University of Global Health Equity Institutional Review Board (Ref: UGHE-IRB/2024/286) on 12/03/2024 and Neno District Health Research Committee and National Health Sciences Research Committee (approval number 4404) on 29/03/2024. Participation in the study was voluntary and written informed consent was obtained from all participants. Identifiers were used to protect participant identity.

### Reflexivity statement

Faith and Abraham were the principal investigators for this study. Faith is a Nigerian who had never visited Malawi before and does not speak Chichewa, the local language of Neno District. She has limited knowledge of the district’s traditions, culture, and adolescent contraception issues, despite having implemented various adolescent sexual and reproductive health (SRH) programmes. To address these limitations, she worked closely with Abraham throughout the study, particularly during study design, data interpretation and contextualisation of the findings. Abraham is a Malawian who speaks Chichewa, understands the district’s traditions, and has worked on projects aimed at improving adolescents’ access to contraception.

## Results

Table 1 describes the participant demographics and data collection methods for this study. A total of 56 participants were engaged through 18 in-depth interviews (IDIs) and 4 focus group discussions (FGDs). The sample included adolescents (10 IDIs), parents/guardians (4 FGDs, *n* = 38), healthcare workers and volunteers (4 IDIs), and religious and traditional leaders (4 IDIs).

**Table 1.** Participant demographics and data collection methods

Category	Gender	Data collection method (IDI/FGD)
Adolescents (15-19 years)	5 Male, 5 Female	10 IDIs (5M, 5F)
Parents/Guardians	15 Male, 23 Female	4 FGDs (total *n* = 38)
Healthcare Workers/Volunteers	3 males, 1 female	4 IDIs
Religious Leaders	3 Male, 0 Female	3 IDIs
Traditional Leaders	1 Male, 0 Female	1 IDI
**Total**	**27 Male, 29 Female**	**18 IDIs, 4 FGDs**

The analysis generated three primary themes: (1) A climate of restriction, (2) agency and support, (3) the paradox of acknowledged risk and sustained resistance. These themes were analysed through a rights-based socio-ecological framework, reflecting the complex interplay of factors across individual, interpersonal, community, and health system levels that constrain or enable the realisation of adolescent SRHR. A summary of these key findings across each level of the framework is presented in Table 2.

**Table 2.** Summary of key findings through a rights-based socio-ecological lens

Socio-ecological level	Key barriers (rights gaps)	Key facilitators (rights enablers)	Adolescent preferences
Individual	Fear, shame, misinformation, negative personal experiences with side effects.	The agency is exercised through education and a desire for safe sex.	Discrete, long-acting methods (implants, injectables); peer-based information.
Interpersonal	Gendered power imbalances limit negotiation; parental and partner opposition.	Supportive peers, partners, and some parents.	Service provision via trusted, private community agents (e.g. YCBDAs).
Community	Stigma, judgmental attitudes, and cultural/religious norms prohibiting adolescent sexuality/contraception.	Community campaigns, some religious/cultural support for contraception to avert poverty.	Youth clubs/corners as safe, non-stigmatising spaces for SRH information.
Health system	Judgmental providers, lack of privacy, commodity stock-outs.	Welcoming, confidential services and flexible, adolescent-friendly policies.	Services decoupled from main clinics to ensure privacy and reduce shame.

### Theme 1: a climate of restriction: stigma, misinformation, and systemic barriers

This theme encompasses community and health system-level factors that create a hostile environment for adolescent contraceptive use, directly infringing on rights to privacy, non-discrimination, and information.

#### Judgment and stigma as social control

Adolescents, especially girls, faced pervasive stigma. Fear of being labelled “promiscuous” by community members and providers was a major deterrent. A healthcare worker's comment, “Why are you here? You are too young,” exemplifies judgmental provider attitudes that violate adolescents’ right to acceptable, non-discriminatory care. This stigma was reinforced by some parents and community leaders who viewed contraception as encouraging sexual activity. A parent explained this belief, stating: “*If you give them these things, you are giving them a license to sleep around without fear”* (Parent, FGD 2). This conflation of access with permission illustrates how stigma operates as a tool of social control, actively discouraging service-seeking to enforce community norms around adolescent chastity.

#### An ecosystem of misinformation

Medically accurate knowledge competed with a powerful network of myths that spread rapidly through channels central to adolescent social life. Common false beliefs included contraception causing infertility or cancer, often shared during informal gatherings. Male and female adolescents were exposed to gendered myths; males often cited concerns about reduced potency, while females feared womb damage. These myths, perpetuated by peers, elders, and sometimes through sensationalised stories on social media and radio, actively undermined the right to evidence-based information. A common refrain among adolescents, highlighting peer-to-peer transmission, was: “*I heard from friends that the implant can travel in your blood to your heart and kill you”* (Female adolescent, IDI 03). The persistence of these myths points to a critical gap in engaging adolescent communication networks with credible, countervailing information.

#### Health system shortfalls

While structural barriers like long travel distances and commodity stock-outs affect all clients, their impact is particularly acute for adolescents, amplifying existing fears of stigma. Specifically, the stock-out of adolescents’ preferred discreet contraceptive methods, like injectables, posed a significant barrier. Additionally, the limited availability of peer providers, with whom both male and female adolescents felt more comfortable and preferred as a source of information, was frequently cited as a deterrent, as adolescents of this group value confidentiality. These systemic failures represent gaps in the obligation to provide accessible, acceptable, and quality health services. An adolescent described how the lack of privacy heightened their sense of exposure and shame: “*The room is not closed properly, just a curtain … People outside can hear everything you are discussing with the nurse. You know your business will be the topic of gossip before you even leave*” (Male adolescent, IDI 07). For adolescents, these are not just inconveniences but critical failures that directly contravene their right to confidential care and deter uptake.

### Theme 2: agency and support: navigating pathways to contraceptive uptake

This theme highlights individual and interpersonal-level facilitators that enable adolescents to exercise their SRHR, despite restrictive environments.

#### Exercising agency through education and risk perception

Adolescents demonstrated an ability to exercise agency when they could weigh concrete risks. This was most evident when their desire to protect themselves from pregnancy and sexually transmitted infections was linked to a clear future goal, such as completing their education. This internal cost–benefit analysis often overrode social fears. One adolescent’s rationale captured this deliberate calculus: “*I am not ready for a child, and getting one would mean dropping out of school. That risk is greater than any fear of the injection*” (Female adolescent, IDI 09). This illustrates a pragmatic form of agency where adolescents, when equipped with an understanding of consequences, can make proactive health decisions to safeguard their life plans.

#### Knowledge was a key facilitator

Knowledge acted as the crucial bridge between risk perception and actual service uptake. Adolescents who received accurate information from trusted, adolescent-friendly sources such as peer-led youth clubs, targeted radio programs, or supportive YCBDAs were not only better informed but also felt empowered to act. This access to reliable information bolstered their confidence to navigate a hostile health system. As one participant explained, contrasting knowledge with myth: “*After the youth club session, I understood that the myths were not true. Knowing the real facts gave me confidence to go to the clinic*” (Male adolescent, IDI 04). While sub-theme 2.1 focuses on the internal motivation (*why* they might want contraception), this subtheme highlights the external catalyst (*how* they overcome barriers to get it), showing that accurate information is a necessary tool for translating intention into action.

#### Critical interpersonal support

In a context of widespread opposition, isolated sources of support became lifelines. Support from trusted peers, cooperative partners, or a rare supportive parent was crucial for overcoming hesitation. Notably, some healthcare workers and YCBDAs) consciously positioned themselves as allies by upholding confidentiality, thus directly enabling adolescents’ right to care without mandatory parental consent. A community health worker described their supportive, rights-based approach: “*I tell them, ‘This is your body and your future. My role is to give you the right information so you can protect yourself.’ I never ask for a parent if they come alone*” (Healthcare Volunteer, IDI 16). This demonstrates how individual providers can create pockets of safety within a restrictive system, validating the adolescent’s autonomy.

### Theme 3: the paradox of acknowledged risk and sustained resistance

This theme presents the study's most potent finding, a profound contradiction between community awareness of the harms of early pregnancy and persistent opposition to adolescent contraception.

#### Shared recognition of severe consequences

A consensus existed across all participant groups, adolescents, parents, and leaders, on the devastating socio-economic and health impacts of teenage pregnancy. The consequences of school dropout, unsafe abortion, maternal mortality, and entrenched poverty were clearly and frequently articulated. A parent’s stark comment summarised this acute, pragmatic awareness: “*When a girl falls pregnant, it is not just a child; it is the end of her education and the start of lifelong poverty for her*” (Parent, FGD 1). This universal recognition established a common ground of concern, yet, as the next subtheme reveals, it was insufficient to shift entrenched normative positions.

#### The persistence of opposition

Despite this awareness, cultural and religious norms valuing early fertility and proscribing premarital sex often overrode pragmatic health and wellbeing concerns. This created a rights conflict between the adolescent’s right to health and information and community cultural and religious beliefs. The result is that communities, while recognising the economic risk of pregnancy, choose to accept it over the perceived social and moral risk of sanctioning adolescent sexuality through contraception access.

This tension was also evident among male adolescents whose views were also contradictory. Despite being aware of the socio-economic and health consequences of early pregnancy, they narrated social expectations of their fertility as a masculine marker. One participant said “*We know and see how challenging it is when you have a child especially while still in school, but we are expected to show that we are real men by getting girls pregnant*” (Male adolescent, IDI 07). Another participant said “*I knew early pregnancy causes many problems, but my friends mocked me for staying in a relationship for a long time without getting my girlfriend pregnant, and that is how I ended up having my first child*” (Male adolescent, IDI 10). A religious leader further explained this tension, stating: “*We know the dangers, yes. But giving them contraceptives is like approving what is forbidden, and that would destroy our moral fabric. The pregnancy is a consequence, but the sin comes first*” (Religious Leader, IDI 14). This paradox underscores that interventions must engage with deeply held cultural logics, not just provide services.

## Discussion

This study, framed through a rights-based socio-ecological lens, provides a nuanced understanding of the multi-level factors influencing contraceptive uptake among adolescents in rural Malawi. The findings highlight the complex interplay between awareness of the health and socio-economic consequences of early pregnancy and the cultural, religious and health system factors that shaped attitudes and decisions around adolescent contraception. While many participants recognised the importance of preventing early pregnancy, these considerations were often outweighed by social norms and systemic barriers, limiting adolescents' access to and use of contraceptives. Crucially, the socio-ecological model illuminates how barriers at one level amplify those at another. For instance, community-level stigma directly enables interpersonal-level opposition from parents and partners, which in turn exacerbates individual-level fear and shame, while health system-level failures in privacy and confidentiality validate and compound these social fears.

### Navigating stigma, misinformation, and systemic failures: a rights-based analysis

Consistent with studies across sub-Saharan Africa,^7,10^ adolescents in Neno face a climate of stigma and judgment across ecological levels, directly violating their rights to privacy, non-discrimination, and confidential healthcare. The fear of being labelled “promiscuous” by providers, parents, and community members acts as a potent social control mechanism, discouraging service-seeking. This finding aligns with work in Lilongwe^8^ but is critically extended here by explicitly linking community-level stigma to a health system-level failure to provide adolescent-responsive, rights-based care. For adolescents, the lack of private consultation spaces and judgmental provider attitudes are not just service deficiencies; they are systemic validations of the social stigma they fear. When a clinic’s physical layout allows for eavesdropping or a provider’s demeanour is reproachful, the health system itself becomes an active participant in the social policing of adolescent sexuality, thereby institutionalising rights violations.^14,25^ This systemic failure uniquely impacts adolescents, for whom confidentiality is not a preference but a prerequisite for accessing care without social retaliation.

The pervasive community and interpersonal-level ecosystem of misinformation (such as contraception causing infertility) underscores a critical breach of adolescents’ right to evidence-based information. While myths are common in similar contexts,^8,26^ our study highlights their gendered nature and their reinforcement through the interpersonal networks most trusted by adolescents: peers, older siblings, and, significantly, elders within the family and community who are seen as sources of wisdom. This creates an “infodemic” within the very channels adolescents rely on for guidance, eroding agency and informed consent. The cultural insight that unprotected sex is likened to “eating a sweet without its wrapper” powerfully encapsulates a community-level norm that trivialises long-term health risks, undermining the individual’s right to make safe, informed choices. This network-driven misinformation is particularly potent for adolescents, who are in a developmental stage of seeking identity and validation from their social circles.

### The central paradox: acknowledged harm vs. sustained resistance

A pivotal, cross-level finding is the stark contradiction between widespread recognition of the dire consequences of early pregnancy among participants and persistent resistance to adolescent contraception by parents, community members, religious leaders and healthcare providers. Data reveal that this paradox is co-constructed through the distinct perspectives of different stakeholders across the SEM. While adolescents (individual level) primarily framed risk as personal life disruption, partners and parents (interpersonal level) tended to emphasise the financial burden and added responsibilities, while the community articulated consequences in terms of social standing and family reputation.^27^ Health care workers (health system level) perceived risk in terms of poor health outcomes for both adolescents and newborns, yet judgmental attitudes, lack of privacy and inconsistent adolescent-friendly service delivery discouraged uptake, increasing their vulnerability.^25^ The inclusion of male adolescents was particularly illuminating, as their views frequently embodied the paradox at the interpersonal level. They acknowledged the financial burden of an unplanned pregnancy but simultaneously upheld masculine norms that equated fertility with virility. Religious and traditional leaders (community level), while acknowledging health risks, positioned the moral imperative to uphold cultural sexual norms as paramount.

This presents a fundamental tension: the adolescent’s right to health and bodily autonomy conflicts with deeply held community and religious norms. The paradox suggests that for communities, the tangible economic cost of pregnancy is weighed against, and often outweighed by, the perceived intangible risk of moral decay and social sanction. This norm-based impasse, sustained by the interplay of these multi-level perspectives, explains why informational and service-delivery interventions alone fail. It underscores the necessity for multi-level, dialogue-based interventions that directly engage with these normative frameworks.

### Strengths and limitations

A key strength of this study is its ethical, rights-based implementation, including careful procedures for obtaining assent/consent from minors, which can inform other participatory research with adolescents. The intentional inclusion of male adolescents and multi-level stakeholders provided a uniquely holistic view of the socio-ecological landscape.

This study has several limitations. Perspectives of younger adolescents (15–16-year-olds), who may face heightened vulnerability due to greater parental control, stronger social stigma, and less developed agency, are underrepresented due to unexpected recruitment challenges through our designated community entry points.^28,29^ This limits the full understanding of age-specific barriers within the 15–19 cohort. Second, while the use of young, gender-matched data collectors helped mitigate power dynamics and encourage open discussion, their status as outsiders to the community may have influenced the depth of disclosure on sensitive topics.^30^ However, although this was a qualitative study in just one rural district, the study provides rich, transferable insights into the socio-ecological barriers to adolescent SRHR in similar conservative, resource-limited settings. Lastly, although the findings were interpreted through a rights-based lens, the interview guides were not specifically developed to explore human rights concepts in depth. As a result, rights-related issues emerged through participants’ experiences and were interpreted alongside the socio-ecological framework during analysis, which may have limited exploration of some rights-based dimensions. Future research should incorporate data collection approaches that more explicitly explore adolescents' sexual and reproductive rights.

### Implications for policy and practice

Table 3 outlines specific, actionable recommendations.

**Table 3.** Comprehensive recommendations for a rights-based adolescent contraception strategy in Malawi

Level of action	Recommended action	Key actors/accountable systems
Health system and policy	Mandate and fund the full implementation of adolescent-friendly service standards (privacy, confidentiality, non-judgmental care) in all facilities. Decentralise service provision through strengthened community-based distribution (YCBDAs) and youth corners. Integrate comprehensive SRHR education**,** addressing myths and rights, into school and community curricula.	Malawi Ministry of Health (MOH), District Health Offices, Christian Health Association of Malawi (CHAM), Partners in Health (PIH)
Community and norms	Sustain structured dialogue with religious and traditional leaders to reinterpret norms supporting adolescent health. Launch community-led campaigns featuring positive testimonials to counter stigma and misinformation. Empower male adolescents as partners in SRHR through targeted education programs.	District Council, Traditional Authorities, Faith-Based Organisations, Community Leaders, NGOs
Legal and accountability	Publicly uphold and disseminate existing policies protecting adolescents’ rights to confidential SRH services. Establish clear accountability mechanisms **(**e.g. feedback systems) for adolescents to report rights violations by providers.	Malawi Human Rights Commission, Ministry of Gender, MOH, Legal Aid bodies
Research and monitoring	Conduct longitudinal studies on the impact of community dialogue interventions on normative change. Implement robust monitoring of adolescent contraceptive uptake and rights indicators within the health management information system	Academic Institutions, MOH Monitoring and Evaluation Division, NGOs

To effectively increase adolescent contraceptive uptake in restrictive settings like Neno, stakeholders must address the complex socio-ecological system revealed in this analysis.

(1) *For health systems and policymakers:* There is a need to implement differentiated, rights-based service models. Policymakers must mandate and fund the operationalisation of adolescent-friendly health service standards beyond superficial labels. This requires structural investment to create genuinely private consultation spaces and reliable supply chains for preferred discreet methods, like implants and injectables. Crucially, policy must explicitly protect adolescents’ right to confidential care, legally affirming that parental consent is not mandatory and providing training and supportive supervision for providers to shift from judgmental to rights-based attitudes. Furthermore, our data strongly support the formal integration and scale-up of community-based distribution through trusted Youth Community-Based Distribution Agents and youth clubs as complementary, low-threshold channels. National health policies should create a framework to train, supply, and remunerate these community-based providers, effectively decoupling essential SRH services from the stigmatising environment of main clinics.

(2) *For programme designers and NGOs:* Reframe engagement from information to normative dialogue. Programmes must pivot from standalone information campaigns to facilitated dialogue that addresses the central paradox. This involves designing structured intergenerational forums that bring together adolescents, parents, and community/religious leaders. The goal is not to lecture but to use the shared concern for adolescents’ futures – a point of consensus – as a foundation to critically examine how current norms inadvertently exacerbate the very risks (poverty, school dropout) all parties wish to avoid. Messaging should shift from “contraception prevents pregnancy” to “contraception protects futures, families, and community well-being,” aligning with communal values. Additionally, peer education must be strengthened to equip adolescents not just with facts, but with communication and negotiation skills to navigate power dynamics with partners and elders.

(3) *For research and advocacy:* Target the key gatekeepers shaping adolescent contraception decisions. Our findings demonstrate that resistance to adolescent contraception was reinforced by the attitudes of parents, religious and traditional leaders, some healthcare providers, and male partners. Advocacy efforts should therefore move beyond treating the community as a single audience and instead tailor engagement to these specific groups. At the community level, advocacy should support structured dialogue with parents and community leaders to address misconceptions around adolescent contraception while building on the shared recognition of the consequences of early pregnancy. Religious and traditional leaders should be engaged to identify culturally and socially acceptable approaches that promote adolescents' health and wellbeing without undermining community values. At district and national levels, advocacy should strengthen the voices of adolescents, healthcare providers and district implementers in policy discussions to ensure that programmes and policies reflect the realities experienced in the communities. Future research should also examine how changes in community norms, provider attitudes and adolescent-provider interactions influence contraceptive uptake, rather than focusing solely on service utilisation.

## Conclusion

This study provides a multi-level analysis of the barriers, facilitators, and preferences influencing adolescent contraceptive uptake in rural Malawi. It reveals a profound disconnect between widespread recognition of the severe risks of early pregnancy and persistent socio-cultural and systemic resistance to adolescent SRH services. Key actionable priorities emerging from this rights-based analysis are: (1) transforming health systems to guarantee confidential, adolescent-responsive care as a non-negotiable standard; (2) initiating structured, sustained dialogue with religious and community leaders to reconcile cultural values with adolescent health rights; and (3) scaling up decentralised, youth-preferred service delivery through trusted community-based agents. Future research should evaluate the impact of such multi-pronged interventions on normative change and adolescent SRHR outcomes. Ultimately, overcoming the entrenched barriers documented here requires a committed, rights-affirming approach from all stakeholders, health systems, communities, and policymakers to ensure adolescents can exercise their fundamental right to make safe, informed decisions about their sexual and reproductive lives.

## Data Availability

All the data and documents used during the study are stored in a secure University of Global Health Equity online drive, which will be preserved for 10 years according to the UGHE data policy until it can be disposed. The data can only be accessed after seeking approvals from the institution’s appropriate authorities.
